# Stigmatization related experience among mothers of children with autism

**DOI:** 10.3389/fpubh.2026.1746420

**Published:** 2026-03-09

**Authors:** Otilia-Rodica Butiu, Delia-Andreea Nechifor, Teodora Popescu, Rebeca-Isabela Molnar, Adriana Mihai

**Affiliations:** 1 Pediatric Neurology and Psychiatry Clinic, County Mureş Hospital, Târgu Mureş, Romania; 2 George Emil Palade University of Medicine, Pharmacy, Science, and Technology of Târgu Mureş, Romania; 3 Psychiatry Clinic 1, County Mures Hospital, Târgu Mureş, Romania

**Keywords:** autism, caregiver, Caregiver Burden Inventory, stigmatization, stress

## Abstract

**Introductions:**

This study investigates the relationship between stigmatization and stress levels among mothers of children with autism spectrum disorder (ASD).

**Methods:**

Using items from standardized psychometric tools, the study explores how emotional burden, social strain, and coping strategies contribute to parents' perceived stigma.

**Results:**

Findings suggest that indirect measures of stigma through psychological assessment offer a more comprehensive understanding than direct questioning, which may elicit guarded responses.

## Introduction

Autism Spectrum Disorder (ASD) is defined as a neurodevelopmental disorder that has its first manifestation in early childhood and is characterized by persistent communication difficulties, and repetitive behavioral patterns, presenting specific interests and activities (1). According to DSM-5-TR, the diagnosis of autism spectrum disorder requests the presence of the following criteria: the enduring lack of social communication and interaction, limited and repeated patterns of behavior, interests or activities, early outbreak in developmental period, symptoms that intervene with daily functioning and are not better explained by other conditions (2). Taking into account the high prevalence rate of children diagnosed with autism in the last 20 years and the observation of delay in initiating specific treatment, it seems that the specialists in the field of ASD have gained a new niche of study such as stigma among relatives of autistic patients (3).

According to Oxford Learner's Dictionaries, stigma represents negative feelings that people have about particular circumstances or characteristics that somebody may have (4); in case of patients with ASD, negative feelings about the chaotic behavior of the children with autism, their rigid routines and passions (3). Stigma is described by Erving Gottman in his book (5) as a relationship between attribute and audience, which leads to being excluded and discredited from social integration. It represents a mark that often causes the others to inflict negative beliefs on a person perceived as different, to persist in these beliefs and to adopt an exclusion or avoidant behavior towards the <<marked>> person (6). There are 4 categories of stigma: the first one of them is public stigma, the situation when the degradation of a person is encouraged by the negative perception of specific social characteristics. The second one is self-stigma, which focuses on harmful convictions about the self; it originates from the person's knowledge of the devaluation of the traits he/she possesses. Another type is represented by stigma by association and it concentrates on individuals involved in formal or informal relationships with a stigmatized person. The last type is structural stigma and it manifests as a form of discrimination specific to societal institutions and structures which promotes stigmatizing perception through their adopted measures (ex: not allocating enough funds for the psychiatric field) (7, 8). This study does not focus on the individuals living with autism, but on their caregivers. On one hand, the caregivers understand the disorder and how it affects the daily functioning of the children. On the other hand, they also understand the verbal and non-verbal clues in social communication, making them more vulnerable to criticism, rejection and shame because of the disorder of the children. Therefore, the caregivers find themselves in a delicate position, which brings to them a lot of stress and erosion to their mental health.

They are subjected to what is known as courtesy stigma, stigma by association or family stigma, a type of stigma which focuses on the internalization of negative attitudes directed not to themselves, but to their children with autism in this case. They may start to feel incompetent, being the subject of stigma for their “lack” of discipline over their mentally ill child. Their self-image starts to alter due to diminished sense of purpose and value, low self-esteem and self-efficacy (9). Being the witnesses of their children's stigmatization, the caregivers (in this case the mothers) empathize with the outcast status of the autistic children and start feeling ashamed, sad or guilty for their condition. Moreover, the great amount of care they need to take adds a lot more stress and exhaustion, sometimes leaving the mothers to deal with symptoms of anxiety and depression and then to find a method of coping with all the situations (8).

When the stress of parents of children with autism is discussed, it is relevant to take into consideration their ability to manage the stress. There are previous studies focusing on their educational level (5), socioeconomic status and age (6). This study emphasizes how stigma and parenting children with ASD induce stress which can shape the mental health status of the caregivers, knowing that they are associated with elevated levels of depression, distress and anxiety (7).

The prevalence of autism spectrum disorder (ASD) has seen a marked global increase in recent decades, with current estimates ranging from 0.02% to 3.66% of the population (8). Mothers of children with ASD often encounter significant psychosocial challenges, one of the most pervasive being social stigma (9).

In Romania, recent trends suggest a decline in stigmatization, potentially due to three main factors: an increase in specialized psychotherapeutic services for children with ASD, state recognition of the condition as a severe disability, which grants official support and a rising public awareness due to media figures and parents who have openly discussed the challenges of raising children with autism (10). These changes appear to coincide with a decrease in divorce rates and greater involvement of fathers in caregiving roles (11, 12).

This study investigates the relationship between stigmatization and stress levels among mothers of children with ASD. Using standardized psychometric tools, the study explores how items from tests reveal mothers' perceived stigma. Findings suggest that indirect measures of stigma through psychological assessment offer a more comprehensive understanding than direct questioning, which may provide avoidant responses. We used items from psychological tests which address stigma (items from Caregiver Burden Inventory, Depression, Anxiety, and Stress Scales (DASS) and Strategic Approach to Coping Scale (SACS)).

## Materials and methods

We evaluated in our study 41 mothers of children with autism spectrum disorder who were at the Pediatrics Neuropsychiatry Clinic Targu Mures during the period of May–August 2025, using a questionnaire with general sociodemographic questions. Participants completed the following standardized instruments: Caregiver Burden Inventory (Novak and Guest 1989), Depression, Anxiety, and Stress Scales (DASS) and Strategic Approach to Coping Scale (SACS). From the previously mentioned tests we selected all the questions related to stigmatization related experience for better understanding stigma and not to engage in direct asking about it, but to make the profile of a caregiver of children with autism disorders and her needs. We chose from these tests the items which were related with stigmatizations for better understanding stigma and not to engage in direct asking about stigma, but to make a portrait of a caregiver of children with autism disorders and her needs. Before filling the items described above, the mothers completed a form which requested demographic data in order to help us design the portrait of the caregiver of a child with autism spectrum disorder (age, living area zone, marital status, number persons per family, number children per family, educational level) and questions related to the medical background of their children (Do you know the General Practitioner?, age group distribution of children with autistic disorder, Are you conscious of the diagnosis of your children?, Have you been informed of the diagnosis of your children? years from receiving the diagnosis, Do you know the specific recommendations?)

Data was collected from two Romanian ASD outpatient and inpatient clinics, from caregivers of children and adolescents with an ASD diagnosis that has been confirmed by experienced clinicians, ICD-10 diagnoses (F84.0/F84.1/F84.5).

Informed consent was obtained from all participants, and the study was approved by the Ethics Committee of the Mureş County Clinical Hospital (No. 8318/26.06.2025). The analysis focuses on items from each instrument that specifically relate to stigmatization, including emotional reactions, interpersonal relationships, and coping behavior. For the statistical analysis, the GraphPad InStat software was used. The Student's t-test and the Chi-square test were applied to determine associations. Continuous variables were statistically summarized as mean ± SD (standard deviation). All tests were two-tailed, and statistical significance was considered at a threshold of α ≤ 0.05.

## Results and discussions

A total of 41 mothers of children diagnosed with ASD were recruited from the Pediatric Neurology and Psychiatry Clinic in Târgu Mureş. All the collected data can be found in Table 1. It needs to be added that parents who know their General Practitioner have a good collaboration with him and all of them declare that they have a strong relationship with the medical staff and a positive influence when it comes to communication and support. Moreover, those mothers who know the specific recommendation for their children describe in most cases going with them to ABA (Applied Behavior Analysis) therapy sessions.

**Table 1:** Demographic data.

**Demographic data**		**Percentage**
Age	20–29 years old	12%
	30–39 years old	48%
	40–49 years old	25%
	50–59 years old	15%
Living area zone	Urban	54%
	Rural	46%
Marital status	Married	68%
	Unmarried	32%
No. Persons/family	2 (single parent family)	10%
	3	32%
	4	45%
	5	7%
	6	3%
	7	3%
No. Children/family	1	30%
	2	47%
	3	17%
	4	3%
	5	3%
Educational level	Higher education	23%
	High school and professional school	77%
Do you know the general practitioner?	Yes	90%
	No	10%
Age group distribution of children with autistic disorder	0–5 years old	32%
	5–10 years old	38%
	10–15 years old	20%
	15–18 years old	10%
Are you conscious of the diagnosis of your children?	Yes	100%
Have you been informed of the diagnosis of your children?	Yes	100%
Years from receiving the diagnosis	0–5 years	60%
	5–10 years	22%
	10–15 years	18%
Do you know the specific recommendations?	Yes	78%
	No	22%

68% of the participating parents were married. Marital status appears to influence stress levels, with lower stress reported among married parents (25 out of 50); however, this finding was not statistically significant (*p* = 0.47). Even if the parents are married, the caregiver is usually the mother. Although it may seem easier to take care of a child because the spouse helps, the difference is not significant. The “Caregiver Burden Inventory” is offered to assess the caregiver's statements — in our case, the parent caring for a child with ASD.

**Figure 1:**
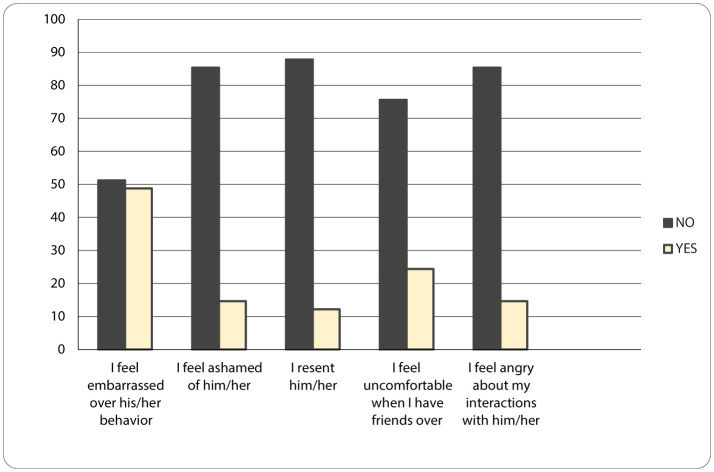
Emotional Impact (CBI) graphic (no – if the mother chose a response < 3, she does not identify with the statement, Yes – if the mother chose a response ≥ 3, she identifies with the statement).

**Figure 2:**
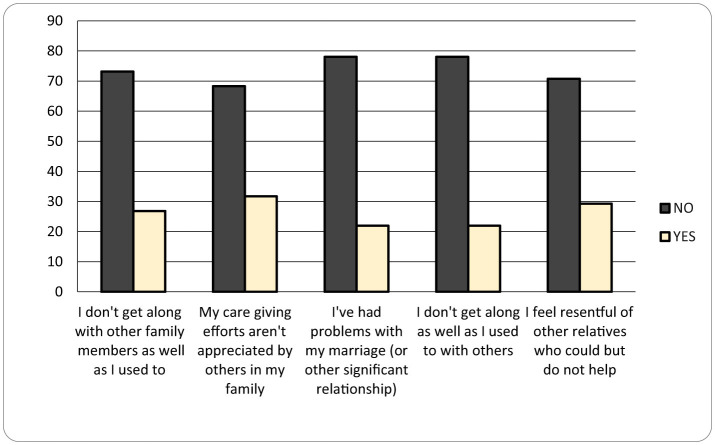
Social strain (CBI) graphic (no – if the mother chose a response < 3, she does not identify with the statement, yes – if the mother chose a response > 3, she identifies with the statement).

As seen in Figures 1 and 2, **Emotional Impact (CBI)** are reflected by the following sentences: “I feel embarrassed because of my child's behavior” (46%), “I feel ashamed of him/her” (15%), “I feel like rejecting him/her” (12%), “I feel uncomfortable when friends visit” (24%), “I feel angry when interacting with him/her” (15%). Stress level is higher when it comes to the behavior of the children with ASD which affects the life of the entire family. Moreover, it was observed from mothers' interviews that the stress level was more elevated when they left the family environment, because the children were becoming more agitated and frustrated. Thus, mothers reported judgement, non-acceptance and guilt, because these types of situations correlate with the assumption of bad parenting by other people and stigma related experience toward the mothers (13–15). It is important to see that mothers recognize the feeling of rejecting their child only in 12% of cases, they are more embarrassed of child behavior in public, experiencing uncomfortableness in social situations - when friends come to visit them. In this case, they seem to be ashamed of the judgment of their friends. On the other hand, friends may feel petty for the mother, for the child disease (it is a human reaction even in chronic diseases, but it is not helpful) (12). As a consequence, the friends of the mothers rarely come to visit her and the behavior is not proper (16).

Caregiver Burden Inventory (Novak and Guest 1989) was used to assess the caregivers of patients with dementia in the first place. The test includes 24 items that evaluate different aspects of caregiver strain. The inventory measures five distinct dimensions of the caregiver burden- time dependence burden, developmental burden, physical burden, social burden and emotional burden. Each item is rated on a 5 point Likert scale (from 0=not at all to 4 – very descriptive) (15, 16) In order to be significant, the test result needs to be above 36 (15). There are a few versions of the test which evaluate the burden or the stress of parents caring for children with ASD. Niekerk et al. used the Caregiver Burden Inventory to evaluate the burden of those caring for children with ASD (17). For example, Vilanova et al. used the “Zarit Burden Interview,” a variant of “The Caregiver Burden Inventory” adapted into Brazilian Portuguese (18), to assess parental stress in caring for children with ASD.

We found in our study that for about a quarter of mothers the score of Caregiver Burden Inventory is above 36 and they feel overwhelmed and stressed. It is important to test parents and when using this test in clinical work, we can talk with parents about every item which scores above 3, even if the score is below 36.

Lately, there are studies which assess the effective intervention for parents whose child is autistic and who exhibits symptoms of depression, anxiety or stress. The most effective therapies like we see in the meta-analysis (19) were Mindfulness-Based Interventions (MBIs), Acceptance and Commitment Therapy (ACT), Psychoeducational Interventions (PED), and a combination of Mindfulness and Parent-Mediated Maladaptive Behavior Interventions as the most effective approaches. MBIs enhance present-moment awareness and foster self- compassion, which helps manage unpleasant thoughts and improves parent-child interactions (20, 21). Mindfulness-based psychoeducational interventions have been shown to be more effective than parent mediated interventions targeting core symptoms (PIM-CS), which focus more on managing children's behavioral problems rather than providing immediate stress relief for parents. This underscores the importance of tailored mental health interventions for parents in the context of child-focused interventions. According to SUCRA values, Psychoeducational Interventions (PED) had the highest relative effect on parental self-efficacy, though neither PED nor parent-mediated maladaptive behavior interventions showed significant improvement over usual care. Systematic reviews indicate that well-designed psychoeducational interventions can enhance parents' knowledge and skills about autism (19).

This is in accordance with our findings, because the mothers are primarily ashamed of the behavior of their child in public (46%), not of their children as a person (only 15% of mothers feel ashamed of their children). Psychoeducation gives them a feeling of competence, of understanding and accepting the child. From the clinical experience of working with parents of children with autistic spectrum disorder before and during the conduction of this study, psychoeducation has proved to be very effective even for the father, knowing the steps of the recovery plan and what he is supposed to do to help the child. If both participate at sessions, they are more likely to support each other. This underlies the importance of psychoeducation for the parents, the acceptance of the disorder (rather than concentrating on irrational thoughts) and focusing on the present moments.

**Social Strain (CBI)** is mentioned in the following sentences: “I do not get along with other family members” (27%), “My caregiving efforts are unrecognized by my family” (32%), “I have marital problems” (22%), “I feel disconnected from others” (22%), “I feel resentment toward relatives who could help but don't” (29%). In this situation, there are more problems in the family, even if the family members try to help, maybe their efforts are not enough for the main caregiver. Taking care of the child may take all day long. When the disease is associated with mental and language delay, the caregiver has to supervise all the activities of the child: 16–18 h of work/day or 12 h if they go to school, and this is going to exhaust the mother. Even if she gets help, she may not recognize that help. Many mothers say that it is very important for the children to go to special schools or associations to receive therapy and ignore the fact that it is important to have some hours in which they do not have to take care of children. Most mothers are the only caregiver of the children, representing a decisive factor of their personal lives from which they become deprived, having a low emotional state, feeling desperation, overwhelming, frustration because of the lack of help from the husband or the family (22, 23).

Most studies on family burden in children with ASD focus on mothers, who typically serve as the primary caregivers. Consistent with our findings, fathers are generally less involved in daily household tasks, attending primarily to responsibilities outside the house. However, mothers manage the more demanding aspects of care, ensuring that the child's basic needs are met, which often results in higher levels of burden and the tendency to place others' needs above their own (18, 24, 25). Previous research also suggests that the elevated stress experienced by mothers of children with ASD arises from the necessity of fulfilling multiple roles to meet the demands associated with the condition (26).

These items are relevant for the stigma inside the family and community stigma. The results are relatively high. A quarter of the mothers feel disconnected from the family members, lack of help and understanding; they feel rejected from communities. Through the process of research, there had not been found a study about the efficacy of family therapy in the stigmatization related experience of parents with children diagnosed with autistic disorders, but it is important that psychoeducation should include both members of the family, husband and wife, and, if it is possible, the grandparents could help with taking care of the child (in Romania it is happening) and could also be included in sessions of psychoeducation to understand the condition.

**Figure 3:**
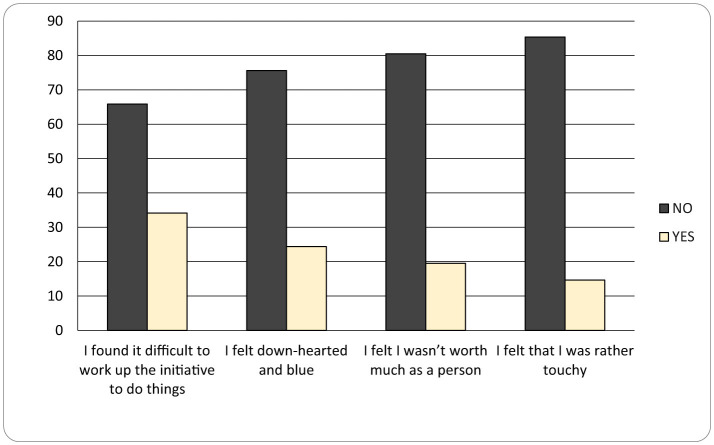
Psychological Symptoms (DASS) graphic (no – if the mother chose a response < 2, she does not identify with the statement, yes – if the mother chose a response ≥ 2, she identifies with the statement).

As seen in Figure 3, **Psychological Symptoms (DASS)** are revealed through the following sentences: “I have been in situations so distressing that I felt relief when they ended” (34%), “I felt down and anxious” (24%), “I was in a state of nervous tension” (20%), “I felt I had little value” (15%). These sentences show psychological symptoms, the frequency of symptoms of anxiety and depression being really high - 20–25%. Some mothers are treated for depression or anxiety and the others who exhibit symptoms ignore them.

Exposure to courtesy stigma may lead to internalizing stigmatizing beliefs by the mothers, which in turn can contribute to the development of psychological distress. This may manifest through anxiety—characterized by fears concerning the child's future behavior, apprehension about the child's wellbeing when the mother is no longer able to provide care or after her death, and negative cognitive appraisals of the future—as well as through symptoms of depression and elevated stress levels.

The Depression Anxiety Stress Scales – 21 Items (DASS-21) is a self-report instrument designed to assess three related negative emotional states: depression, anxiety, and stress. It is a shorter version of the original 42-item DASS and consists of 21 items, with 7 items per subscale. It is not a diagnostic tool but provides an indication of symptom severity in the three domains. Respondents rate the extent to which each statement applied to them over the past week on a 4-point Likert scale ranging from 0 (“Did not apply to me at all”) to 3 (“Applied to me very much or most of the time”). Subscale scores are summed and multiplied by 2 to match the DASS-42 scoring (27–29).

Parents of children with ASD, particularly mothers, generally report moderate to high levels of stress, elevated anxiety, and mild to moderate depression on the DASS-21. The elevated scores reflect the psychological burden associated with caregiving and are consistently higher than in parents of neurotypical children (9, 30, 31).

In the 1970s, it was relatively common for mothers of children with autism to be labeled as “refrigerator mothers,” a term implying that parents failed to provide sufficient affection or warmth to their children. This belief was widespread among child psychiatrists and within society at a large scale and it represented a significant burden and a source of stigma for families. Nowadays it is well established that parenting style or educational approach does not cause autism spectrum disorder (19). Autism is currently understood as a complex condition with both genetic and maybe epigenetic determinants (possibly involving the activation of certain genes under specific environmental conditions). Nevertheless, some parents perceive this explanation as stigmatizing, seeming to imply a sense of personal responsibility for transmitting the disorder to their child.

**Figure 4:**
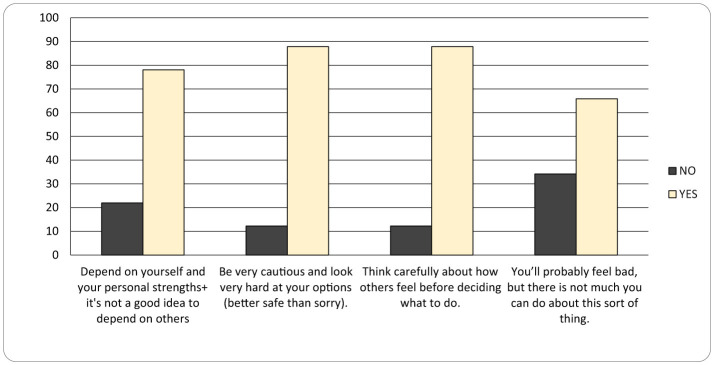
Coping Strategies (SACS) graphic (no – if the mother chose a response < 3, she does not identify with the statement, yes – if the mother chose a response ≥ 3, she identifies with the statement).

As seen in Figure 4, **Coping Strategies (SACS**): “I rely on myself because I don't believe in depending on others” (78%), This item in coping is very high and the caregiver feels that she can rely only on herself, because the others do not have time and the skills to deal with children with autistic disorder. Living with this coping is very difficult. One hypothesis would be that it is a learned coping mechanism, because it is difficult to take care of a child with autism disorders. In our clinical experience we noticed that if the mother wants to find medical help for a child's dental problem, it is very hard in the area to find a dentist who can manage this problem without performing a general anesthesia. When the children are sent for medical problems, mothers find it very difficult to go to medical doctors; some of them confess that there is a stigmatization related experience from the medical staff. Many of them come to consultations, even to short term hospitalizations in the Child and Adolescence Psychiatry section, but some find it very difficult to go in another medical setting.

“I am cautious and evaluate options carefully” (88%): The stress level is higher when it comes to the behavior of autistic children going outside the family environment, because the child becomes more agitated and the mother more sensitive to the negative attitude of the others. Mothers attentively choose whether to go or not to social gatherings, because of the public opinion (neighbors, acquaintance etc.) which labels them and their children. They think a lot about incidents that can happen when they are not physically near them. Moreover, one major source of stress for the female caregivers is the future of the child, his/her possibility to continue the educational path, to have a job, to form a family and to be independent. Thus, mothers are caught in an internal conflict between their desire for their children to lead a normal life, their understanding of the judgement of the society and the fear of losing control over their autistic children (24).

“I consider others' feelings before making decisions” (88%) is also a mechanism that reveals the attention to other people's reactions, sometimes even sensitivity to other people's reactions. Because our study focuses on women, it needs to be taken into consideration that women are educated and more sensitive to human relations and interactions.

“I feel bad in such situations, but there is little I can do” (66%). This is a very important theme that shows little control over a child's behavior. A review which evaluated the quality of life of parents of children with ASD in comparison with the parents of healthy children describes a lower social, physical, psychological and spiritual level of quality of life among parents of children with ASD compared to the high level of quality of life of parents with healthy children. The protective factors are represented by the educational level and the level of severity of ASD of the child (13).

The Strategic Approach to Coping Scale (SACS) is an instrument developed by Stevan E. Hobfoll and colleagues which is grounded in the Conservation of Resources theory and the multiaxial model of coping. The scale assesses behavioral coping strategies in a social context, focusing on concrete actions taken when facing stress or adversity. The explored dimensions are:

- Active vs. passive coping mechanism.
- Prosocial vs. antisocial management of social resources.
- Direct vs. indirect focus toward others and the stressor.

In its common form it contains of 52 items organized into 9 subscales (e.g., Assertive Action, Avoidance, Social Joining, Seeking Social Support, Cautious Action, Instinctive Action, Indirect Action, Antisocial Action, Aggressive Action) (19, 32, 33). The SACS has been adapted into Romanian language and cultural context, with studies examining its factor structure, reliability and validity (34).

“There is always a feeling of regret, compassion and longing for him when he is not home. However, we wish for his emotional state to improve, even if we experience a sense of guilt when he is hospitalized without us, his parents. We try not to worry too much and look for beneficial solutions to support and understand our child” – it represents the statement of a mother whose 14-year-old adolescent son was hospitalized in our clinic for multiple aggressive episodes and he needed physical and medical coercion. This can be another stigma related experience. This is not the norm in our clinic. When it comes to dealing with the severe aggressivity of teenager with autism spectrum disorders, this is not the norm in our clinic. When it comes to dealing with the aggressivity and the perpetual movement of children with ASD, we prefer to use either pharmacological coercion such as the combination between Haloperidol and Diazepam intramuscular administered in emergency, after that a combination of pharmacology treatment or non-pharmacological techniques in teenagers. We believe that they need to be taught through behaviors that do not inspire fear on them and avoid the association that the hospital represents a place filled with torture and brutality (14).

Our patients with autism come to us from a very young age and because it is a chronic disorder, we also know their family very well and we can send the mother to receive psychological or psychiatric help if they need it. We send children for therapies and we know the mothers are stressed, with anxiety and depression; but it was a surprise how much they feel that “I rely on myself because I don't believe in depending on others” (78%). I think it is about the repetitive failure of our system to help these mothers, even though stigmatization, in combination with unprocessed and socially unrecognized grief.

**Figure 5:**
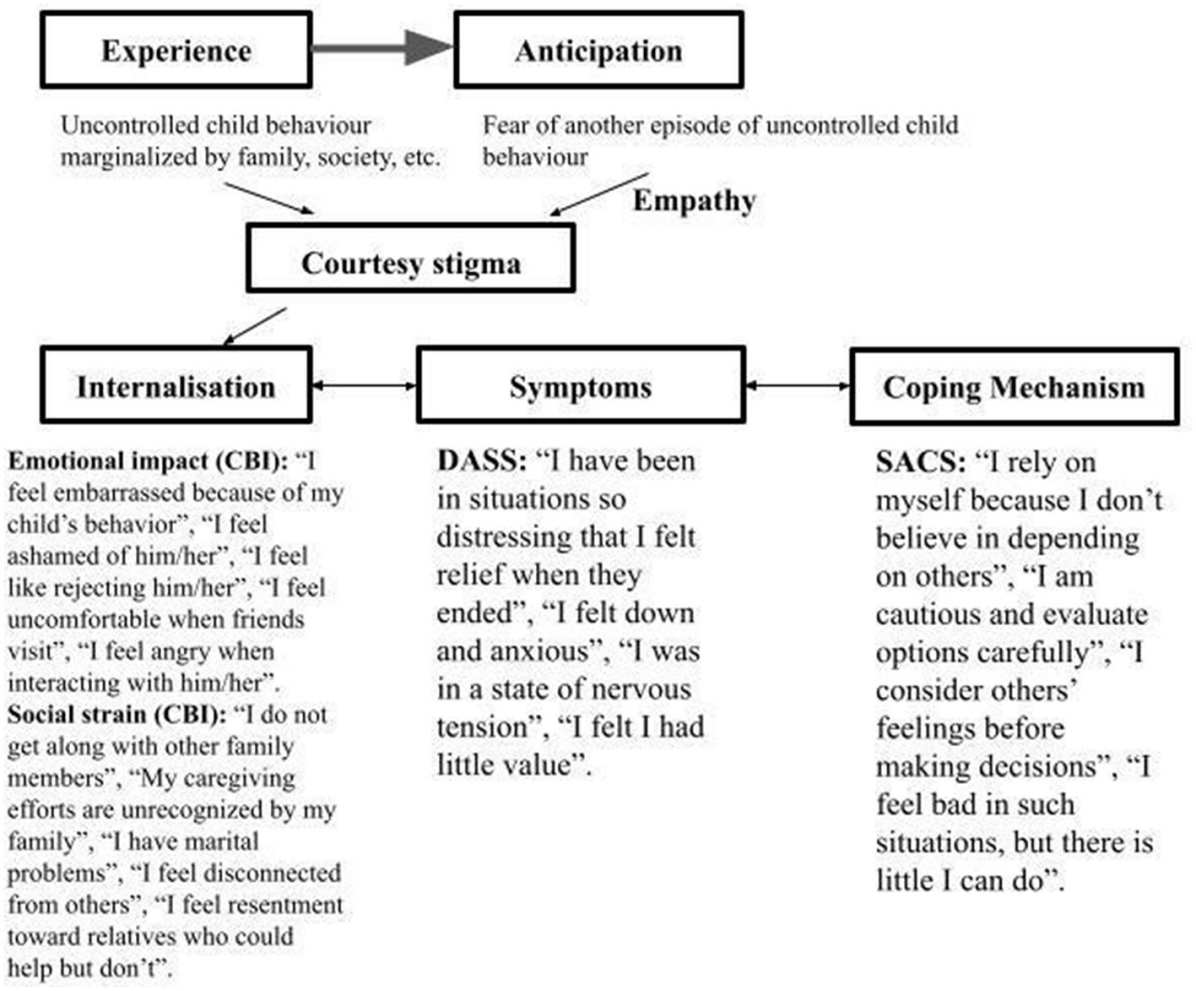
The mechanism of stigmatization – related experience among the mothers in this study.

As seen in Figure 5, we prepared a scheme that summarizes our study and how we have observed the mechanism of stigma among the mothers in our sample. It was adapted from The model of the circle of stigma by Mitter et al. (6). The scheme summarizes how parents' experiences of caring for a child with autism spectrum disorder (ASD) become part of a stigma-driven psychological process. Initial experiences of socially unpredictable child behavior expose parents to negative social reactions, including judgment, blame, and consecutively to exclusion (5–7). These experiences converge in anticipatory stigma, a perpetual state in which caregivers develop a fear of future behavioral incidents and upcoming social disapproval, reinforcing vigilance and emotional strain. Through repeated exposure, parents become subject to courtesy (affiliate) stigma, internalizing public stigma directed at their child and integrating it into their own self-concept (5, 9, 12). Empathy for the child intensifies this process, as caregivers often absorb responsibility and shame in an effort to protect the child from social harm (3, 11, 15).

As illustrated in the scheme, affiliate stigma is subsequently internalized, giving rise to emotional responses such as embarrassment, shame, guilt, and self-blame, alongside social consequences including withdrawal, family conflict, and perceived lack of recognition or support (3, 9). These internalized stigma processes are closely linked to psychological distress, including feelings of anxiety, devaluation, and emotional exhaustion, which function as downstream manifestations of sustained stigmatization rather than isolated mental health outcomes (8, 16). In response, caregivers adopt coping mechanisms that often emphasize self-reliance, emotional suppression, or cautious social engagement, reflecting attempts to manage stigma exposure rather than resolve caregiving demands (9, 12). The scheme englobes the process of how exposure to stigmatizing reactions of the public for autistic child behavior can translate to a constant fear of rejection and lack of parental control, shaping a poor self-image of the mother and degrading mental health. Thus, mothers learn how to lean only on themselves, how to hide their emotions or how to avoid social gatherings (6–8).

### Study limitations

This study was exclusively conducted with Romanian mothers, which may limit the generalizability of the findings to other cultural contexts. Cultural factors and traditional social roles may have influenced the experiences and perceptions of stigma reported by participants. Additionally, the sample size was relatively small, with only a limited number of mothers involved, which may reduce the statistical power and the representativeness of the results. Future research should include larger and more culturally diverse samples to confirm and extend these findings.

### Study originality

Using selected items from established psychological tests to assess stigmatization experienced by mothers of children with autism spectrum disorder represents an original approach. Organizing these items into specific categories allows for a structured analysis of different dimensions of stigma, facilitating a more nuanced understanding of the social and psychological challenges faced by these mothers.

## Conclusions

Our findings suggest a strong association between stigmatization and elevated stress levels among mothers of children with ASD. Using validated psychological instruments that contain stigma-related items provides a more nuanced understanding of this phenomenon than direct questioning alone. This method allows for a richer analysis of how stigma interacts with emotional burden, social support, and individual coping mechanisms. Being the main caregiver, it is important to assess interventional services for supporting the mothers' needs, because when we help the mothers, we also help the child and increase her capability to sustain the child. If the mother does not receive help and support from the family, she cannot access supportive social services and medical services for her child (35–38).

Furthermore, it is important for the family to participate together in sessions of psychoeducation focused on understanding the disorder, learning to accept the child's stereotypical behaviors in public, and developing appropriate strategies for managing aggressive behavior. Families should also be informed that new or unfamiliar environments may induce stress in the child and be encouraged to recognize and appropriately address such situations. In addition, collective efforts are needed at a societal level to reduce and destigmatize the prejudice directed toward mothers of children with autism spectrum disorder (ASD).

## Data Availability

The original contributions presented in the study are included in the article/supplementary material, further inquiries can be directed to the corresponding author.
